# The Text4HealthyAging Program: An Evidence-Based Text Messaging Innovation to Support Healthy Urban Aging in Canada and Australia

**DOI:** 10.1177/23337214221081378

**Published:** 2022-03-01

**Authors:** Ejemai Eboreime, Arto Ohinmaa, Benjamin Rusak, Keri-Leigh Cassidy, Jason Morrison, Patrick McGrath, Rudolf Uher, Sandra Meier, Marie-Josee Fleury, Srividya N. Iyer, Soham Rej, Frances Batchelor, Pazit Levinger, Christa Dang, Malcolm Hopwood, Francis N. L. Acquah, Janet Dzator, Gail Tomblin Murphy, Jordan Warford, Lori Wozney, Isabelle Vedel, Jacqueline Gahagan, Olga Theou, Prosper Koto, Tara Sampalli, Susan Kirkland, Nicholas Watters, Vincent I. O. Agyapong

**Affiliations:** ^1^ Department of Psychiatry, Faculty of Medicine and Dentistry, 12357 University of Alberta, Edmonton, AB, Canada; ^2^ School of Public Health, College of Health Sciences, 70411 University of Alberta, Edmonton, AB, Canada; ^3^ Department of Psychiatry, Faculty of Medicine, 3688 Dalhousie University, Halifax, NS, Canada; ^4^ Department of Psychology & Neuroscience, Faculty of Science, Dalhousie University, Halifax, NS, Canada; ^5^ IWK Health Centre, Halifax, NS, Canada; ^6^ Department of Psychiatry, McGill UniversityMontreal, QC, Canada; ^7^ 110764 National Ageing Research Institute, Parkville, VIC, Australia; ^8^ Department of Psychiatry, 213116 University of Melbourne, Parkville, VIC, Australia; ^9^ Positive Wellness Recovery Centre, VIC, Australia; ^10^ Newcastle Business School, 5982 University of Newcastle, Australia; ^11^ Mental Health and Addictions, Nova Scotia Health Authority, Halifax, NS, Canada; ^12^ Department of Family Medicine, 12367 McGill University, Montreal, QC, Canada; ^13^ 3684 Mount Saint Vincent University, Halifax, NS, Canada; ^14^ Geriatric Medicine Research, 432234 Nova Scotia Health Authority, Halifax, NS, Canada.; ^15^ School of Physiotherapy, 3688 Dalhousie University, Halifax, NS, Canada; ^16^ Department of Community Health & Epidemiology, 3688 Dalhousie University, Halifax, NS, Canada; ^17^ 434957 Mental Health Commission of Canada, Ottawa, ON, Canada

**Keywords:** aging, community, mental health, public health/public policy

## Abstract

Age-friendly cities are crucial to achieve the WHO goal of healthy aging. Such cities promote opportunities for health, participation, and security, thus enhancing quality of life as people age. Older people commonly experience psychosocial challenges such as anxiety, depression, substance abuse, loss of autonomy, grief, fear, and loneliness. Australian and Canadian cities continue to seek innovation to improve healthy urban aging and create more age-friendly environments for older adults. There is increasing evidence on the effectiveness and feasibility of mobile technology in health promotion and closing psychological treatment gaps. Older adults have been demonstrated to engage frequently with mobile devices, particularly text messaging. In this article, we conceptualize the Text4HealthyAging, an evidence-based text messaging innovation to support healthy urban aging in Canadian and Australian cities.

## Introduction

The population is aging in both Canada and Australia. As of 2016, approximately 15% of Canadians and Australians were aged 65 years and above. This figure is projected to reach about 20–22% by 2036(Australian Bureau of Statistics, 2019; Shooshtari et al., 2020). According to the Organization for Economic Co-operation and Development (OECD) in 2015, 43% of the older population resides in cities(OECD, 2015). As modern cities rapidly evolve infrastructurally and socially, there is a need to incorporate healthy urban aging into routine services and city development. The World Health Organization (WHO) defines healthy aging as “the process of developing and maintaining the functional ability that enables wellbeing in older age”(Rudnicka et al., 2020).

Age-friendly cities (AFCs) are crucial to achieve the WHO goal of healthy aging. Such cities promote opportunities for health, participation, and security, thus enhancing quality of life as people age(Jeste et al., 2016; Plouffe & Kalache, 2010). Some studies show that older adults living in urban environments tend to experience more psychological distress than those residing in rural communities(Alcaniz et al., 2020; Michel, 2020; Wu et al., 2017). Perceived socioeconomic difficulties (such as the absence of family relationship and family care as well as loss of social support among) among urban dwelling seniors have been attributed to this outcome. Further, cities and densely populated areas tend to be associated with noise, agitation, as well as other social and environmental stressors(Alcaniz et al., 2020; Michel, 2020). Common psychological issues affecting older people may include, but are not limited to, anxiety, depression, and substance abuse. Common social and emotional issues may involve loss of autonomy, grief, fear, loneliness, financial constraints, and lack of social networks(Abraham Cottagiri et al., 2021; Roe et al., 2020; Werth et al., 2002). A complex relationship exists between psychosocial distress and cognitive and physical health that is important to address given the increasing prevalence of multi-morbidity in older age(Sakib et al., 2019).

The ongoing COVID-19 pandemic compounds mental stress and health-related problems as well as existing physical, financial, and social barriers to accessing health care. Studies reveal that the pandemic has intensified post-traumatic stress injuries, anxiety, depression, suicidal ideation, and sleep disorders among residents of municipalities in Australia and Canada(Agyapong et al., 2021a, 2021b; Brydon et al., 2021; Dawel et al., 2020; Lawal et al., 2021; Nkire et al., 2021; Osiogo et al., 2021; Sapara et al., 2021; Shalaby, Adu, et al., 2021; Pachana et al., 2020). Psychosocial support is needed to motivate older people to adhere to medical and psychological prescriptions, as well as physical activities(Levinger et al., 2021). Thus, enhancing supportive environments wherever they live is essential for healthy aging. Older people in Canada and Australia increasingly describe experiencing social isolation. About 30% of the older population are at risk of becoming socially isolated(Barbosa Neves et al., 2019; Sipowicz et al., 2021). In addition to those living at home, 2.6% and 6% of Canadians and Australians aged 65+ reside in residential aged care facilities, respectively(Dyer & Tilden, 2021; Statistics Canada, 2018), the majority of which are located in urban settings(Colman, 2021; Hanlon & Halseth, 2005; Williams & Kulig, 2012). There is evidence that living in a long-term care facility can contribute to feelings of loneliness and social isolation, as well as sedentary living(Barbosa Neves et al., 2019; Parry et al., 2019). Social isolation in older people, irrespective of where they reside, increases all-cause mortality by 50%(Holt-Lunstad et al., 2010).

Staying socially connected, increasing physical activity, healthy eating, taking steps to minimize their risks for falls and other healthy behavioral and environmental adjustments can help older adults live longer and healthier lives(Jeste et al., 2016; Parry et al., 2019).

In many urban settings, older adults experience challenges with accessing health services, recreational facilities, social, and religious gatherings(Sato et al., 2019). Socio-ecological models indicate that physical activity among older adults is influenced by individual as well as environmental characteristics and the interactions between them(Hallal et al., 2012; Van Cauwenberg et al., 2018).

Efforts to achieve age-friendly cities will benefit from innovative intervention packages that mitigate the effects of physical, psychosocial challenges and multiple morbidities, and promote healthy aging among seniors. There is a growing interest in the use of mobile health (mHealth) technologies to create healthy psychosocial environments for older adults within their communities. The application of mHealth to support healthy aging is now a priority area for policy development in many jurisdictions, including Canada and Australia(Matthew-Maich et al., 2016). This concept paper proposes how a supportive text messaging innovation for older people can bridge this gap.

### The Text4HealthyAging Program

The Text4HealthyAging program is an adaptable, evidence-based, supportive messaging innovation, designed to promote healthy aging among people aged 65+ in Canadian and Australian cities. With the help of mental health therapists, psychologists and people with lived experiences, our not-for-profit partner, the Global Psychological eHealth Foundation (GPEF), developed a bank of supportive text messages that are based on principles of cognitive behavioral therapy. Over 500 messages have been developed which include general supportive messages aimed at motivation, health promotion, and providing health resources through embedded links to websites, pictures or videos, emergency numbers, and education about COVID-19 vaccines and pandemic control measures. Supportive text messages have also been adapted to address people’s various mental health diagnosis and follow the earlier development of versions that support other population groups such as young people (MoreGoodDays), veterans and first responders (Text4PTSI and Text4Well-being), as well as people experiencing the COVID-19 pandemic (Text4Hope), adult anxiety and depression (Text4Mood), and natural disasters (FMMStrong), among others. (Please see: https://www.resiliencenhope.org/). These messages are designed to promote healthy aging as well as provide supportive treatment for psychosocial disorders. However, all subscribers receive the general supportive messages, people with specific needs are able to subscribe to receive messages tailored to specific diagnosis such as post-traumatic stress disorder, frailty, or sociocultural environment (e.g., language and gender-related messages). An example of these supportive messages is:

• *My life during retirement will not be immune from difficulties, but I know I will have peace even during difficult times. I will overcome the storms by connecting with friends and family and staying positive.*

Building on the evidence of efficacy, effectiveness, cost-effectiveness, and acceptability of these supportive text messaging programs when utilized to bridge the psychological treatment gap for mental health patients, as well as promote healthy living across various population groups, we conceptualized an adaptation of this intervention service for the aging population in Canadian and Australian cities. The text4HealthyAging will be adapted to meet the diverse, evolving needs of older people. For example, the messages will provide psychological and educational support for seniors who have diabetes, arthritis, cognitive impairment, depression, anxiety, as well as provide general health promotion and useful resources (such as describing what action to take or who to contact when faced with common challenges in their city of residence). The messages will also provide links to information on neighborhood features and community programs for older adults.

The existing messages will be adapted (and new targeted messages developed) by a team of mental health professionals, geriatric specialists, dieticians, physiotherapists and exercise scientists, social workers, representatives of older people, and gender experts, and experts from municipal public health. There will be opportunities for adaptations to fit cultural contexts (e.g., First Nations/indigenous populations in Canada and Australia), different gender identities, occupational exposures/experiences (e.g., veterans), the bereaved, multiple language needs, etc. Currently, existing messages have been translated into French, Arabic, Punjabi, and Mandarin.

### Delivery Platform

The GPEF has also developed a platform to deliver these text messages across the globe. Please see: https://www.resiliencenhope.org/programs. About 10% of subscribers to our Text4Hope program (Agyapong et al., 2021), a population-based intervention providing support to Albertans during the COVID-19 pandemic, are aged 60 years and above. This figure closely mirrors the seniors population of Alberta (11%)(Statistics Canada, 2016). Like other age categories, seniors found the Text4Hope program relevant, and felt the program made them feel connected to the health system despite the constraints of the pandemic(Shalaby, Vuong, et al., 2021). Building on this, the Text4HealthyAging presents an opportunity to promote general wellbeing (physical, psychological, and social) and provide a supportive environment for older people, particularly during the COVID-19 pandemic, and aligned with the goals of AFCs.

### Sex and Gender-Based Considerations

Given the evidence that gender sensitivity is important to promote acceptability and satisfaction with supportive text messaging programs (Shalaby, Vuong, et al., 2021), both sex and gender-based considerations will be incorporated throughout program design, implementation, and evaluation of the Text4HealthyAging. The bank of supportive text messages to be used in the intervention will be reviewed by our sex and gender expert group, to ensure that content of the messages include intersecting sex and gender-based considerations and are appropriate for the target population at each site. We will use the 2021 Canadian Institutes of Health Research’s Sex and Gender-Based Analysis Plus (SGBA+) analytic framework to evaluate the impact of the interventions in relation to sex and gender specific indicators. Specifically, we will ask participants to rate a random selection of supportive text messages to determine the influence of sex and gender in their rankings. This will help to further develop the text message bank for specific diverse sexes and genders.

### Is There Scientific Evidence in Support of the Efficacy and Effectiveness of Supportive Text Messaging Programs?

There is evidence that supportive text messaging can significantly improve the psychosocial well-being of both healthy individuals and people with health conditions. Supportive text messaging can be remotely delivered, is scalable, economically reliable, convenient, and there is growing evidence of applicability in mental health (Adu et al., 2021; Agyapong et al., 2021; Hall et al., 2015; Shalaby et al., 2021). In three randomized controlled trials conducted in Ireland and Canada (Agyapong et al., 2017; O’reilly et al., 2019), people with Major Depressive Disorder (MDD) who received twice-daily supportive text messages had significantly greater reductions in their depression symptom scores than people who received the usual treatment. In one of these trials, the mean difference in change of Beck Depression Inventory (BDI)-II scores, between the intervention and the control group, was −7.9 (95% CI −13.06 to −2.76, Cohen’s d = 0.85) in favor of the intervention group after 3 months (O’Reilly et al., 2019). Further, 83% of subscribers to the Text4Mood program implemented in Alberta reported that the daily messages contributed to improving their overall mental wellbeing(Agyapong et al., 2016).

An evaluation of the Text4Hope program also showed that participants who had received daily supportive text messages for only 6 weeks had significantly (*p* < .005) lower prevalence rates in the 2 weeks preceding data collection for (a) moderate/high stress (78.8% vs. 88.0%), (b) likely Generalized Anxiety Disorder (GAD) (31.4% vs. 46.5%), (c) likely MDD (36.8% vs. 52.1%), and (d) suicidal ideation/thoughts of self-harm (16.9% vs. 26.6%), compared to new subscribers who were yet to receive any daily supportive text messages (Agyapong et al., 2021). There was also demonstrable 23% reduction in the mean anxiety symptom scores for Text4Hope subscribers after 3 months (Agyapong, Shalaby, et al., 2021). We anticipate similar outcomes with the Text4HealthyAging program as well.

### Is it Feasible to Implement Supportive Text Messaging Programs for Older Adults?

Supportive text messaging for the urban older adults is feasible to implement in Canada and Australia. Text messaging is available on most cell phones, and does not require software or mobile application downloads, external equipment, fixed lines, or other hardware to function. Mobile phone penetration, as share of population, in Australia and Canada is between 80 and 90%(Governement of Canada, 2018). Over 60% and 85% of older people own basic mobile phones in Canada and Australia respectively(Granwal, 2021; Jacobson et al., 2017; Oliveira, 2014; Statista Research Department, 2015). Generally, it is estimated that 99% of received mobile text messages are opened, and 90% of all text messages are read within 3 minutes of receipt(Johnson, 2013). Contrary to the stereotype that older adults do not frequently engage with technology, data suggest that, compared to smartphone apps, there is no significant difference in the use of basic text messaging across age groups in Canada(Statista Research Department, 2021). In another study, 94% of seniors engage with text messages weekly, sending and receiving an average of 32 messages per month(Mosio, 2021).

### Will Supportive Text Messaging Program be Acceptable to Urban Dwelling Older Adults?

Several studies have reported high user satisfaction of the supportive text message interventions. An evaluation of the Text4Mood and Text4Hope programs found that 80% and 88% of participants, respectively, agreed that asynchronous supportive text messages should be provided during follow-up care, while only about 50% and 70% of participants, respectively, agreed to the use of video conferencing consultations (Agyapong et al., 2013, 2016; Shalaby, Adu, et al., 2021). Related to the plan to use Text4HealthyAging to combat isolation and loneliness among seniors and create health cities, 86% of Text4Hope subscribers reported they felt connected to a support system because of receiving the daily supportive messages (Shalaby et al., 2021; Sipowicz et al., 2021). Inferring from this supporting evidence, we anticipate comparable results among the older adult population in our study sites when the Text4HealthyAging is deployed.

## Conclusion

Canadian and Australian cities continue to seek out approaches to evolve more into AFCs, the use of technology is also being explored. However, there is the general assumption that technology-based interventions may not be very acceptable or feasible to implement among the older adult population, there is sufficient empirical evidence that the older adults are engaging more with technology. The Text4HealthyAging is a feasible solution to improve urban aging as it utilizes basic text messaging services which, as demonstrated by research, is well utilized by older adults in these countries. It is our anticipation that this innovation will be sustainably incorporated to improve the health of this population in Canada and Australia.
